# Effects of citicoline administration on synaptic proteins in rapid eye movement sleep-deprived rats

**DOI:** 10.22038/IJBMS.2022.60756

**Published:** 2022-05

**Authors:** Aysen Cakir, Busra Ocalan, Cansu Cansu, Guldal Gulec Suyen, Mehmet Cansev, Nevzat Kahveci

**Affiliations:** 1 Department of Physiology, Bursa Uludag University School of Medicine, Bursa, Turkey; 2 Department of Pharmacology, Bursa Uludag University School of Medicine, Bursa, Turkey; 3 Department of Physiology, Acibadem Mehmet Ali Aydinlar University School of Medicine, Istanbul, Turkey

**Keywords:** Citicoline, Postsynaptic density, protein-95, REM sleep, Sleep deprivation, Synapsin I, Synaptophysin

## Abstract

**Objective(s):**

Sleep has a pivotal role in learning-memory and sleep deprivation (SD) negatively affects synaptic functioning. Cytidine-5-diphosphocholine (Citicoline) has been known to improve learning and memory functions. Our objective was to explore the effects of Citicoline on hippocampal and cortical synaptic proteins in rapid eye movement (REM) sleep-deprived rats.

**Materials and Methods:**

Rats (n=36) were randomly divided into 6 groups. Environmental control or sleep deprivation was done by placing the rat on a 13 cm diameter platform (Large Platform [LP] group) or on a 6.5 cm diameter platform (REMSD group), respectively, for 96 hours. Rats randomized for controls (Home Cage [HC] group) were followed up in home cages. Rats in each of the REMSD, LP or HC group were randomized to receive either saline (0,9%NaCl) or Citicoline (600 μmol/kg) intraperitoneally twice a day for four days. After the experiments, rats were sacrificed; their cerebral cortices and hippocampi were dissected for analyzing the levels of pre-synaptic proteins synaptophysin and synapsin I, and the post-synaptic density protein-95 (PSD-95) by Western-blotting.

**Results:**

Hippocampal levels of PSD-95, but not the pre-synaptic proteins, were reduced by REM sleep deprivation. Citicoline treatment ameliorated the reduction in PSD-95 levels in REM sleep-deprived rats. On the other hand, REM sleep deprivation was not found to be significantly effective on pre- or post-synaptic proteins in cerebral cortex.

**Conclusion:**

REM sleep deprivation reduces hippocampal PSD-95 levels which are enhanced by Citicoline treatment. These data propose that Citicoline may ameliorate the adverse effects of SD on hippocampal synaptic functioning.

## Introduction

Sleep is an essential part of a healthy lifestyle. Several hypotheses have been proposed to explain the function of sleep. Although there are lots of unresolved questions about its functions, it is widely accepted that sleep plays a major role in body homeostasis and has a pivotal effect on different levels of brain organization (1). Sleep has a critical role in brain maturation and developmental plasticity as well as in ameliorating neuronal networks, which constitute significant parameters for memory consolidation in the hippocampus (2). Sleep is divided into two distinct phases: rapid eye movement (REM) sleep and non-rapid eye movement (non-REM) sleep. REM sleep is characterized by muscle atonia and rapid movements of the eyes. The firing patterns observed in learning are replayed in the REM sleep period, rendering it a fundamental contribution to hippocampus-dependent learning and memory (3). Synaptic plasticity, the most common theory for learning and memory, is known as the changes in synapses’ strength as a response to activity. Long-term potentiation (LTP) or long-term depression (LTD) is responsible for the increase and decrease in synaptic efficacy, respectively. Sleep has been shown to support synaptic plasticity at the molecular level. On the contrary, sleep deprivation adversely affects synaptic plasticity (4) through several mechanisms, including alterations in the molecular composition of synapses (5) and/or disruptions in neurotransmitter release, neuronal activity, and hippocampus-dependent memory (6). 

Hippocampus is a crucial brain area for temporary storage of new information and the formation of memory while permanent storage is provided by broadly distributed cortical networks (7). While negative effects of sleep deprivation on hippocampal synaptic functioning are well established (8), the impacts of sleep deprivation on cortical synaptic structures and/or functions are substantially more ambiguous. For example, the miniature excitatory postsynaptic current amplitude was faintly decreased and intrinsic membrane excitability was increased while miniature inhibitory postsynaptic currents were not affected in prefrontal cortices of mice (9). Another report showed no effect of 8 hr or 48 hr sleep deprivation on mRNA levels of brain-derived neurotrophic factor (BDNF), Synapsin I, and calcium-calmodulin dependent kinase II (CaMKII) in neocortices of rats (10). Besides, 8 hr sleep deprivation was indicated to increase BDNF, activity-regulated cytoskeleton-associated protein (Arc), and tissue plasminogen activator (tPA) mRNA levels while those of matrix metalloproteinase-9 (MMP-9) were decreased in cerebral cortices of rats (11). 

The organization of pre- and post-synaptic proteins regulates the dendritic spine morphology which is significant for synaptic plasticity. Synapsins are a family of pre-synaptic proteins which also modulate synaptic functions by altering synaptogenesis and neuronal plasticity. Synapsin I and II are the major isoforms in neurons (12). Synapsin I is a neuron-specific phosphoprotein tethering to the actin-based cytoskeleton which regulates the pre-synaptic network (13). Regulation of synaptic vesicle fusion kinetics by synapsin phosphorylation increases synaptic vesical availability for neurotransmitter release (14) and allows synaptic vesicles to be transported into the ready-release pool (15). In addition, synaptophysin, another presynaptic protein, plays a major role in synaptic vesicle recycling (16) and regulates activity-dependent synapse formation (17). Post-synaptic density protein 95 (PSD-95), is a scaffolding protein that is located at the post-synaptic membrane. PSD-95 is bound to several proteins on the cellular membrane such as ion channels, cell-adhesion molecules and receptors (18), kainate, and N-methyl-D-aspartate (NMDA) receptors (19). Therefore, PSD-95 regulates the trafficking and localization of receptors in the membrane (19). 

Cytidine 5-diphosphocholine (Citicoline) is an endogenous nucleotide composed of cytidine and choline (20); it is essential in the synthesis of membrane phosphatidylcholine (PC) via the Kennedy pathway (21). Administration of Citicoline has been documented to exhibit cardiovascular (22, 23), metabolic (24, 25), neuroendocrine (26, 27), and neuroprotective (28-30) effects *in vivo*. Citicoline was indicated to enhance brain PC and other phospholipids’ levels *in vivo* (31).

In a novel study, we showed that Citicoline administration alleviates memory deficit by REM sleep deprivation (32). This finding correlates with previous reports that showed cognitive functional recovery by Citicoline administration under degenerative conditions (33-36).

However, the possible mechanism of action needs to be investigated. Therefore, the goal of the present study was to examine synaptic molecular mechanisms by which Citicoline improves REM sleep deprivation-induced memory impairment. For this purpose, the effects of sleep deprivation as well as Citicoline administration on synaptic proteins were investigated both in the hippocampus and the cortex. 

## Materials and Methods

***Animals and treatment***

Adult (8-12 weeks old) male Wistar albino rats (n=36), weighing 200-300 g, were purchased from Bursa Uludag University Experimental Animals Breeding and Research Center, Bursa, Turkey. Experiments conformed to the NRC Guide for the Care and Use of Laboratory Animals. The study was approved by the Local Ethics Committee on Experimental Animal Research of Bursa Uludag University, Bursa, Turkey (Approval ID: 2019-13/08). The rats were acclimatized to laboratory conditions for 2 days before the onset of the experiments. The rats were randomly assigned to 6 groups: Home Cage group treated with Saline (HC+Saline), Home Cage group treated with 600 μmol/kg Citicoline (HC+C600), Large Platform (LP) group treated with Saline (LP+Saline), Large Platform (LP) group treated with 600 μmol/kg Citicoline (LP+C600), REM Sleep-Deprived (REMSD) group treated with Saline (REMSD+Saline) and REM Sleep-Deprived (REMSD) group treated with 600 μmol/kg Citicoline (REMSD+C600). Citicoline was dissolved in saline. The injections were made intraperitoneally (1 ml/kg) twice a day for 4 consecutive days. The dose of Citicoline was selected on the basis of previous studies which reported beneficial effects of Citicoline on improving memory (32) as well as providing neuroprotection and axon regeneration (29, 30). 

***Induction of REM sleep deprivation***

REM sleep deprivation was performed for 96 hr using the Flower-pot method (37). Animals were housed individually in plexiglass cages (30x23x37 cm) with free access to food and water, under a 12 hr:12 hr light-dark cycle in a temperature-controlled room. Rats in REM Sleep Deprivation groups were placed on 6.5 cm diameter platforms located 2 cm above the water surface in the center of plexiglass cages. The water temperature was adjusted to 22 ± 1 ^°^C. The technique takes advantage of the fact that loss of muscle tone during REM sleep deprivation causes rats to fall into the water to awaken and interrupt the REM phase of sleep. In order to provide controls for the exposure of animals to social isolation and immobilization, rats in the Large Platform (LP) groups were placed on larger platforms with a 13 cm diameter located 2 cm above the water surface in the center of plexiglass cages for the same period (96 hr) of time. The rats in Home Cage (HC) groups were housed in their home cages (3 rats per cage) throughout the experiment. 

***Western blot analysis***

Upon completion of experiments, rats were decapitated under deep anesthesia; the right hippocampus and right frontal cortex from each rat were dissected by the same researcher and immediately homogenized. Each homogenate was placed in a microcentrifuge tube and stored at -80 ^°^C until further use. The homogenates’ total protein contents were analyzed by Bicinchoninic Acid Assay (BCA) method. Equal amounts of protein were loaded and separated by sodium dodecyl sulfate**-**polyacrylamide gel (SDS-PAGE; Mini Protean II, Bio-Rad, Hercules, CA, USA) electrophoresis. Then the proteins were transferred to polyvinylidene fluoride (PVDF) membranes (Millipore, Billerica, MA, USA) and membranes were blocked with 5% non-fat skim milk (Carnation, Glendale, CA, USA). The membranes were incubated overnight with primary antibodies directed against PSD-95 (1:1000; Cell Signaling Technology, Danvers, MA, USA), synaptophysin (1:1000; Sigma, St Louis, MO, USA), and synapsin I (1:1000; Abcam, Cambridge, MA, USA). The next day, the membranes were incubated with the proper HRP-linked secondary antibody (1:5000, Cell Signaling Technology, Danvers, MA, USA) for 1 hr at room temperature. Finally, the membranes were incubated with enhanced chemiluminescence solution (Millipore, Billerica, MA, USA) and optical densities of digital images were analyzed using a digitized scanner (CDigit, LI-COR Biotechnology, Lincoln, NE, USA). The membranes were treated with stripping buffer (Thermo Fisher Scientific, Rockford IL, USA), then the procedure was repeated with mouse anti-β-III-tubulin antibody (1:1000, Cell Signaling Technology, Danvers, MA, USA) and appropriate secondary antibody. Data were expressed as synapsin I/β-tubulin, synaptophysin/β-tubulin, or PSD-95/β-tubulin ratio for each sample.

***Statistical analysis***

Analyses were performed using Sigma Plot version 12.5. Data were expressed as mean±standard error of means (SEM). Groups were compared using One-Way Analysis of Variance (ANOVA) followed by *post-hoc* Tukey test. The statistical significance level was set at *P*<0.05.

## Results

***Effects of citicoline treatment on hippocampal synaptic protein levels in REM sleep-deprived rats***

**Figure 1:**
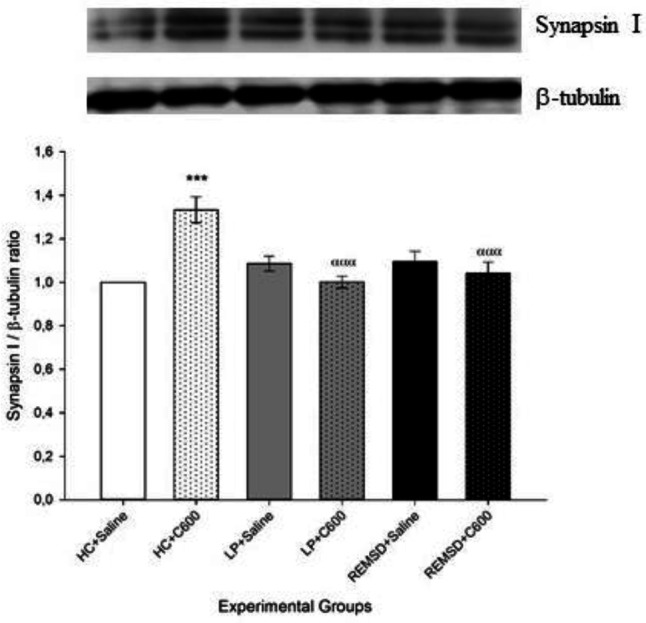
Effects of Citicoline treatment on hippocampal synapsin I levels. Data were expressed as synapsin I/β-tubulin ratio. Hippocampal levels of synapsin I were higher in HC+C600 compared with the HC+Saline group (^***^P<0.001) and were lower in LP+C600 and REMSD+C600 compared with the HC+C600 group (^ααα^*P<*0.001)

Hippocampal levels of synapsin I were higher in Citicoline treated HC rats (HC+C600) in comparison with those treated with saline (HC+Saline; *P*<0.001). Rats in the HC+C600 group also had higher synapsin I levels compared with LP and REMSD rats which received 600 μmol/kg Citicoline (LP+C600 and REMSD+C600, respectively). However, no significant difference was found among saline-treated groups as well as between LP+Saline and LP+C600 groups or REMSD+Saline and REMSD+C600 groups (Figure 1).

**Figure 2:**
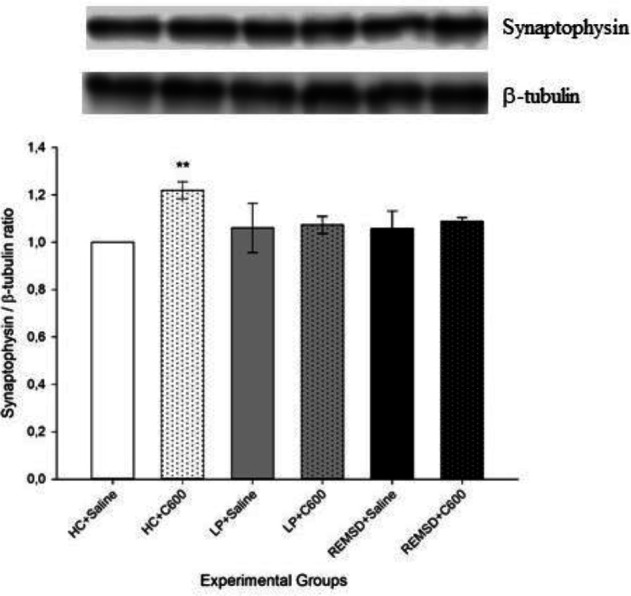
Effects of Citicoline treatment on hippocampal synaptophysin levels. Data were expressed as synaptophysin/β-tubulin ratio. Hippocampal levels of synaptophysin were higher in HC+C600 compared with the HC+Saline group (^**^P<0.01)

 Similar to synapsin I findings, synaptophysin levels were higher in HC rats treated with 600 μmol/kg Citicoline (HC+C600) compared with those treated with saline (HC+Saline; *P*<0.01). No significant difference was found among saline-treated groups as well as between LP+Saline and LP+C600 groups or REMSD+Saline and REMSD+C600 groups (Figure 2).

**Figure 3:**
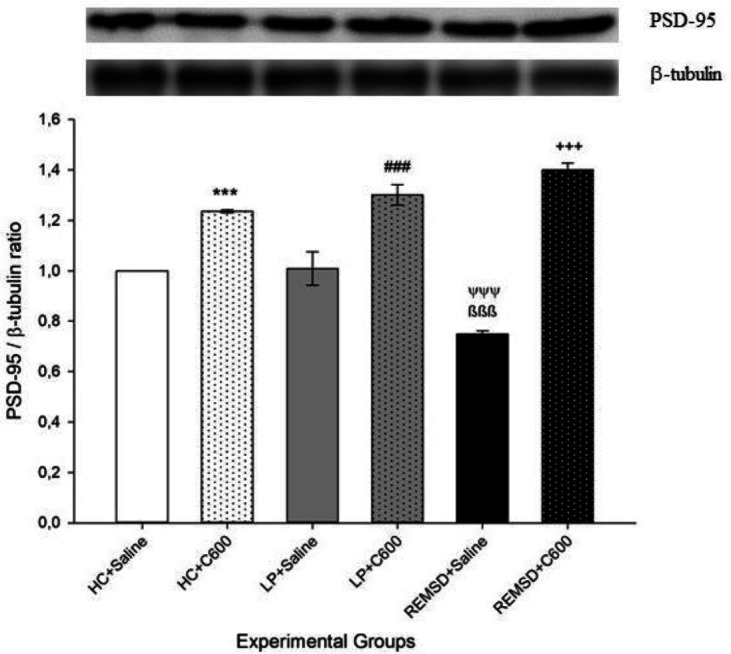
Effects of Citicoline treatment on hippocampal PSD-95 levels. Data were expressed as PSD-95/β-tubulin ratio. Hippocampal PSD-95 levels were higher in rats in HC, LP and REMSD groups which received 600 μmol/kg Citicoline (HC+C600, LP+C600, REMSD+C600; respectively) compared with those treated with saline (HC+Saline (^***^P<0.001), LP+Saline (^###^*P<*0.001) and REMSD+Saline (^+++^*P<*0.001) groups, respectively). Rats in the REMSD+Saline group had significantly lower PSD-95 levels compared with those in the LP+Saline (^ψψψ^ *P<*0.001) and HC+Saline (^ßßß^*P<*0.001) groups

Hippocampal PSD-95 levels were higher in rats in HC, LP, and REMSD groups which received 600 μmol/kg Citicoline (HC+C600, LP+C600, REMSD+C600; respectively) compared with those treated with saline (HC+Saline, LP+Saline, and REMSD+Saline groups, respectively; *P*<0.001). Whereas there was no significant difference between LP+Saline and HC+Saline groups, rats in the REMSD+Saline group had significantly lower PSD-95 levels in comparison with those in the LP+Saline and HC+Saline groups (*P*<0.001) (Figure 3).

***Effects of citicoline treatment on cortical synaptic protein levels in REM sleep-deprived rats***

**Figure 4:**
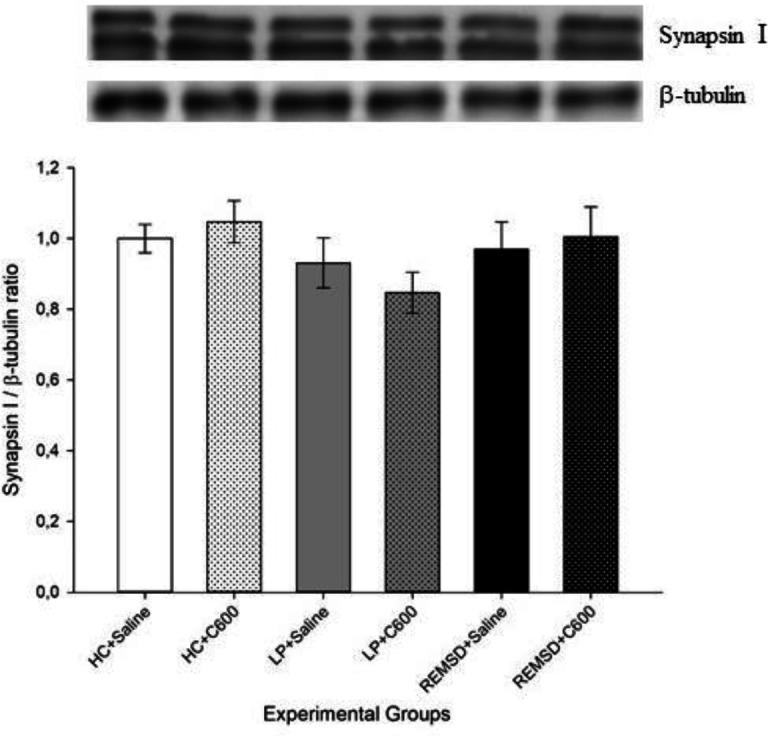
Effects of Citicoline treatment on cortical synapsin I levels. Data were expressed as synapsin I/β-tubulin ratio. No significant difference was found in synapsin I levels between experimental groups

**Figure 5:**
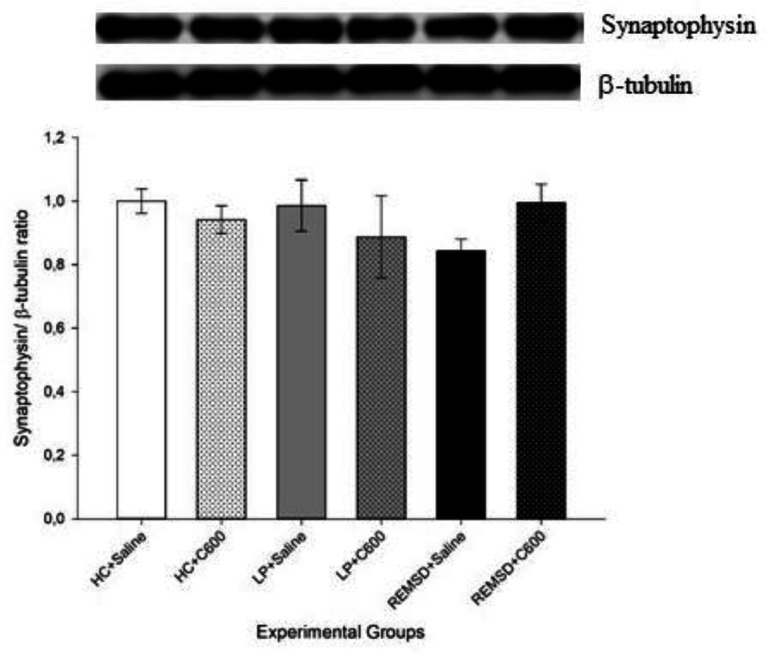
Effects of Citicoline treatment on cortical synaptophysin levels. Data were expressed as synaptophysin/β-tubulin ratio. No significant difference was found in synaptophysin levels between experimental groups

No significant difference was found in synapsin-I (Figure 4) and synaptophysin (Figure 5) levels between experimental groups.

**Figure 6:**
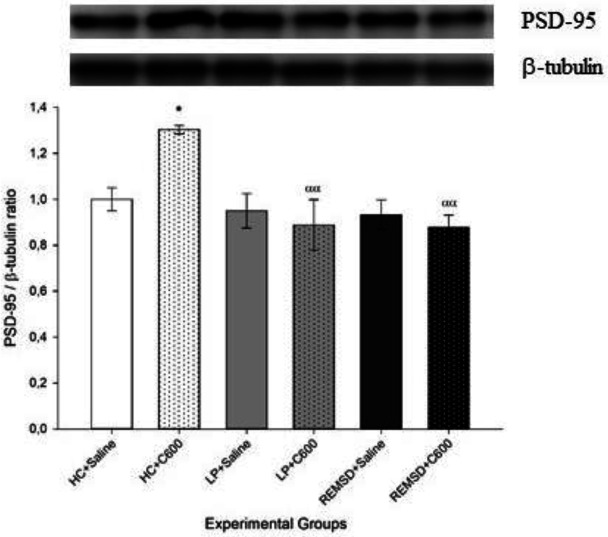
Effects of Citicoline treatment on cortical PSD-95 levels. Data were expressed as PSD-95/β-tubulin ratio. Cortical levels of PSD-95 were higher in HC+C600 compared with the HC+Saline group (^*^P<0.05) and lower in LP+C600 and REMSD+C600 compared with the HC+C600 group (^αα^*P<*0.01)

On the other hand, cortical levels of PSD-95 were higher in HC rats treated with 600 μmol/kg Citicoline (HC+C600) compared with those treated with saline (HC+Saline; *P*<0.05). Rats in the HC+C600 group also had higher PSD-95 levels compared with those in the LP and REMSD groups which received 600 μmol/kg Citicoline (LP+C600 and REMSD+C600, respectively) (Figure 6).

n=6 in each group

n=6 in each group

n=6 in each group

n=6 in each group

n=6 in each group

n=6 in each group

## Discussion

These data show that levels of PSD-95, but not those of the pre-synaptic proteins, synapsin I and synaptophysin, are reduced in rat hippocampus by 96 hr of REM sleep deprivation. The data also indicate that exogenous administration of Citicoline prevents the reduction in hippocampal PSD-95 levels in REMSD rats and enhances the hippocampal levels of both pre- and post-synaptic proteins in the HC group. On the other hand, REM sleep deprivation did not affect pre- or post-synaptic proteins in the cerebral cortex. Hence, our data suggest that REM sleep deprivation diminishes the post-synaptic, rather than the pre-synaptic, structural organization in the hippocampus which is restored by Citicoline treatment. The effect of Citicoline on hippocampal PSD-95 levels might explain, at least in part, the process by which Citicoline prevents cognitive decline by REM sleep deprivation, as reported previously (32).

Sleep is an important event for physical and mental performance (38, 39). Disease, stress, and environmental factors may cause sleep disorders or sleep deprivation. Today, many people are exposed to the negative effects of sleep deprivation due to changing lifestyles. Many studies have focused on alternative treatments that target these negative effects.

REM sleep is a significant phase of sleep for hippocampus-dependent memory. Hippocampus is essential for spatial learning-memory organization and its functions can be altered by modifying synaptic molecular components. Structural changes in neurons and synapses accompanied by new protein synthesis are needed to process new memories (40). Due to these processes of synaptic plasticity, hippocampus-dependent memories are stored for a period of time in the hippocampus and then consolidated in other brain regions, especially in the cerebral cortex. Sleep can modulate expression and levels of such molecules involved in synaptic plasticity as synapsin I, MMP-9, Arc, BDNF, tPA, CaMKII, and cyclic adenosine monophosphate (cAMP) response element-binding protein (CREB) (10,11,41). REM sleep deprivation has deleterious effects on spatial learning and memory (41) through inhibition of long-term potentiation and synaptic plasticity (5). 

The deleterious effects of REM sleep deprivation on synaptic function could have resulted, in part, from consequent alterations in synaptic structural components such as the pre- and post-synaptic proteins. Synapsins and synaptophysin are important pre-synaptic proteins for neurotransmitter release, dendritic spine morphology, and synaptic plasticity (42, 43). Previous studies reported reduced levels of synapsin I after 8- and 48 hr sleep deprivation in the hippocampus but not in the neocortex (10). In addition, synapsin II protein levels were shown to be reduced in the hippocampus by sleep fragmentation (44). 

However, findings on the effect of REM sleep deprivation on pre-synaptic structural components are controversial. For example, in contrast with the above-mentioned reports, a previous study showed that both total synapsin and synapsin I-phosphoSer603 levels increased after 96-hour REM sleep deprivation in the synaptosomes prepared from the whole brain. These findings may explain the reported alterations in neurotransmitter release caused by REM sleep deprivation (45). In addition, hippocampal levels of synapsin I and synaptophysin were found to have not changed in any of the experimental conditions in 96-hour paradoxical sleep deprivation (46). Likewise, no significant alterations in BDNF gene transcription in cortices of long-term sleep-deprived rats for 1 week were detected in another previous study (47). In another study, 8 hr of sleep deprivation was shown to increase BDNF, Arc, and tPA mRNAs in the cerebral cortex, while there was no significant effect on BDNF and tPA expression in the hippocampus (11). We also found no difference with regard to hippocampal pre-synaptic protein levels between control and sleep-deprived rats in this study using the flower-pot method for 96 hr. The discrepancies between these findings may have resulted from different methods and periods that the rats were subjected to sleep deprivation as well as the brain regions investigated and methods used for analyzing pre-synaptic proteins. 

Alterations in levels of post-synaptic proteins have been studied generally with regard to PSD-95, the post-synaptic density protein. Hippocampal levels of PSD-95 were shown to not change in a 96-hour paradoxical sleep deprivation paradigm (46) while we found significant decreases in levels of hippocampal PSD-95 by REM sleep deprivation within the same amount of time in the present study. 

PSD-95 anchors NMDARs to intracellular pathways in order to regulate the strength and plasticity of synapses (18). Hippocampal cell surface expressions of the NR2B subunit of the NMDA receptor (48) and the obligatory NMDA receptor subunit GRIN1 (49) are reduced by sleep deprivation, suggesting an association with the observed reductions in PSD-95 levels in the present study. In addition, sleep-dependent plasticity also involves the contribution of NMDA receptor-mediated CaMKII and ERK phosphorylation (50) since activation of CaMKII has been documented to have a critical role in learning-memory and neuroplasticity in the hippocampus (51). Confirming previous reports (10, 52), a recent study reported the involvement of pCaMKII in REM sleep deprivation-induced memory impairment by demonstrating decreased hippocampal pCaMKII levels after REM sleep deprivation (32). This finding might, as well, be associated with our present observation with regard to decreased PSD-95 levels in the same experimental setting. 

Although the effects of REM sleep deprivation on synaptic structure and functioning have been studied extensively, limited information is available on agents that may ameliorate synaptic dysfunction. In the light shed by recent findings in terms of the beneficial effects of Citicoline on REM sleep deprivation-induced memory decline (32), we further investigated Citicoline’s role in REMSD rats with regard to synaptic proteins. 

Citicoline is essential in the synthesis of membrane phosphatidylcholine (PC) via the Kennedy pathway (21). PC is the most abundant phospholipid in brain membranes, levels of which can be enhanced by precursor supplementation (53). Exogenous administration of Citicoline has also been known to enhance brain levels of PC and other phospholipids *in vivo* (31).

The enhancement in expressions of Synapsin I and PSD-95 is associated with enhanced levels of synaptic membranes, membrane PC, and other phospholipids, as well as increased number of dendritic spines (54, 55). These findings suggest that bioavailability of phospholipid precursors may be involved, at least in part, in the formation of new synapses. 

A number of functional consequences such as enhanced learning and memory in normal rodents (56-58) and recovered motor function in Parkinson’s disease (59) by phospholipid precursor supplementation have been reported previously. Thus, enhanced bioavailability of phospholipid precursors may result in functional benefit through, most probably, enhancing synaptogenesis in the brain (60). 

A recent finding that exogenous administration of Citicoline alleviates REM sleep deprivation-induced memory deficit (32) correlates well with previous reports that showed cognitive functional recovery by Citicoline administration under degenerative conditions (33-35). These findings are supported by the present finding that Citicoline administration enhances the diminished PSD-95 levels due to REM sleep deprivation and are in good accord with significantly increased pCaMKII levels by Citicoline treatment (32). 

## Conclusion

The main outcome of this research is that 96-hour REM sleep deprivation does not affect hippocampal levels of the pre-synaptic proteins, synapsin I and synaptophysin, but reduces those of the post-synaptic protein PSD-95 which are restored by treatment with Citicoline. On the other hand, REM sleep deprivation had no effect on cortical pre- or post-synaptic protein levels. The data support the hypothesis that the hippocampus is more vulnerable to the effects of REM sleep deprivation. In addition, Citicoline treatment also enhances hippocampal synapsin I and synaptophysin levels and increases both cortical and hippocampal PSD-95 levels in control rats. These data suggest that Citicoline may alter synaptic structure under normal conditions and during sleep deprivation, and contributes to the improvement in synaptic functioning, as evidenced by restored memory in REMSD rats.

## Authors’ Contributions

AC, GGS, and MC designed the experiments; AC, BO, and CK performed experiments and collected data; AC, BO, CK, GGS, MC, and NK discussed the results and strategy; NK supervised, directed and managed the study; AC, BO, CK, GGS, MC, and NK final approved of the version to be published; AC, BO, CK, GGS, MC, and NK agreed to be accountable for all aspects of the work in ensuring that questions related to the accuracy or integrity of any part of the work are appropriately investigated and resolved. 

## Conflicts of Interest

The authors declare that no conflict of interest exists.
